# Highlighting the gaps in quantifying the economic burden of surgical site infections associated with antimicrobial-resistant bacteria

**DOI:** 10.1186/s13017-019-0266-x

**Published:** 2019-11-21

**Authors:** Katia Iskandar, Massimo Sartelli, Marwan Tabbal, Luca Ansaloni, Gian Luca Baiocchi, Fausto Catena, Federico Coccolini, Mainul Haque, Francesco Maria Labricciosa, Ayad Moghabghab, Leonardo Pagani, Pierre Abi Hanna, Christine Roques, Pascale Salameh, Laurent Molinier

**Affiliations:** 1 INSERM, UMR 1027, Université Paul Sabatier Toulouse III, Toulouse, France; 2 Epidemiologie Clinique et Toxicologie, INSPECT-LB: Institut National de Sante Publique, Beirut, Lebanon; 3 Department of Surgery, Macerata Hospital, Macerata, Italy; 4 Department of Surgery, Clinique du Levant Hospital, Beirut, Lebanon; 5 Department of Surgery, Bufalini Hospital, Cesena, Italy; 6 Department of Clinical and Experimental Sciences, University of Brescia, Brescia, Italy; 7 Department of Emergency Surgery, Parma MaggioreHospital, Parma, Italy; 8 General, Emergency and Trauma Surgery, Cisanello University Hospital, Pisa, Italy; 9 Unit of Pharmacology, Faculty of Medicine and Defence Health, UniversitiPertahanan Nasional Malaysia (National Defence University of Malaysia), Kuala Lumpur, Malaysia; 10 Scientific committee Global Alliance for Infectons in Surgery, Porto, Portugal; 11 Department of Anesthesiology and Reanimation, Lebanese Canadian Hospital, Beirut, Lebanon; 12 Infectious Diseases Unit, Bolzano Central Hospital, Bolzano, Italy; 13 Infectiousdisease Unit, Sacre CoeurHospital, Beirut, Lebanon; 14 Laboratoire de Génie Chimique (UMR 5503), Département Bioprocédés et Systèmes Microbiens, Université de Toulouse, Université Paul Sabatier, Toulouse, France; 15 Faculty of Pharmacy, Lebanese University, Beirut, Lebanon; 16 Département d’Information Médicale, Centre Hospitalier Universitaire, Toulouse, F-31000 France; 17 INSERM, UMR 1027, Université Paul Sabatier Toulouse III, Toulouse, France

**Keywords:** Surgical site infection, Antimicrobial resistance, Economic burden, Surveillance

## Abstract

Antibiotics are the pillar of surgery from prophylaxis to treatment; any failure is potentially a leading cause for increased morbidity and mortality. Robust data on the burden of SSI especially those due to antimicrobial resistance (AMR) show variable rates between countries and geographical regions but accurate estimates of the incidence of surgical site infections (SSI) due to AMR and its related global economic impact are yet to be determined. Quantifying the burden of SSI treatment is an incentive to sensitize governments, healthcare systems, and the society to invest in quality improvement and sustainable development. However in the absence of a unified epidemiologically sound infection definition of SSI and a well-designed global surveillance system, the end result is a lack of accurate and reliable data that limits the comparability of estimates between countries and the possibility of tracking changes to inform healthcare professionals about the appropriateness of implemented infection prevention and control strategies. This review aims to highlight the reported gaps in surveillance methods, epidemiologic data, and evidence-based SSI prevention practices and in the methodologies undertaken for the evaluation of the economic burden of SSI associated with AMR bacteria. If efforts to tackle this problem are taken in isolation without a global alliance and data is still lacking generalizability and comparability, we may see the future as a race between the global research efforts for the advancement in surgery and the global alarming reports of the increased incidence of antimicrobial-resistant pathogens threatening to undermine any achievement.

## Background

Antimicrobial resistance (AMR) threatens to undermine many advances in the medical field [1] particularly in surgery. Modern medicine is built on the ability of antibiotics to prevent or cure infections [2] but with the growing incidence of AMR added to a dry pipeline [3], we may expect the loss of many advantages in surgical procedures enabled by antimicrobials [4] and a soaring rate of surgical site infections (SSI). Robust data on the burden of SSI show variable rates between countries and geographical regions but accurate estimates of SSI incidence and its related global economic burden are yet to be determined [5]. Quantifying the costs of SSI can inform policy makers about the estimated financial burden of this complication and the cost-effectiveness of interventions to reduce it. Literature review shows that in the absence of a unified epidemiologically sound infection definition of SSI [6–8] and a well-designed global surveillance system, the end result is a lack of accurate and reliable data [9–12]. This can limit the comparability of estimates in terms of rates and costs between countries [13, 14], and the possibility of tracking changes to inform healthcare professionals about the appropriateness of implemented infection prevention strategies. The aim of this review is to highlight the reported gaps in data gathering methodologies and in the evidence-based benefit of some of the current infection control and prevention strategies that limits the possibility of the accurate evaluation of the economic burden of SSI particularly those due to AMR.

## Methods

### Search strategy and eligibility criteria

Search methods for identification of relevant studies was conducted on 10, November 2018, using the below four electronic databases:

- Ovid MEDLINE(R) Epub Ahead of Print, In-Process and Other Non-Indexed Citations, Ovid MEDLINE(R) Daily and Ovid MEDLINE(R) 1946 to Present
- PubMed http://pubmed.gov
- Embase.com https://www.embase.com/#search
- Cochrane Library www.thecochranelibrary.com

The search strategy principle was based on dividing the topic into three concepts: (1) economic burden, (2) surgical site infection, and (3) antimicrobial resistance.

All searches were limited to human and English language with no restriction on age or publication date to ensure that search results include all published articles pertained to the topic.

Ovid Medline was first searched to identify all the possible medical subject headings (MeSH) terms with their corresponding keyword equivalences to increase sensitivity of the search strategy. This technique utilized the many search options available for Ovid Medline such as Boolean operators, truncation, and adjacency searching. The search strategy combined the three concepts as follows: “costs and cost analysis”/or cost-benefit analysis/or “cost control”/or “cost savings”/or “cost of illness”/or health care costs/or direct service costs/or drug costs/or hospital costs/or *health expenditures/or exp economics, hospital/or hospital charges/or exp economics, medical/or fees, medical/or economics, pharmaceutical/ OR cost*.mp. OR ((global or economic* or financial) adj2 (burden* or impact)).mp. [mp = title, abstract, original title, name of substance word, subject heading word, floating sub-heading word, keyword heading word, organism supplementary concept word, protocol supplementary concept word, rare disease supplementary concept word, unique identifier, synonyms] AND exp Surgical Wound Infection/OR (Surg* adj3 wound* adj3 infection*).mp. [mp = title, abstract, original title, name of substance word, subject heading word, floating sub-heading word, keyword heading word, organism supplementary concept word, protocol supplementary concept word, rare disease supplementary concept word, unique identifier, synonyms] OR ((post-surg* or prosthes* or surg* or postsurg* or postoper* or post-opera*) adj3 infection*).mp. [mp = title, abstract, original title, name of substance word, subject heading word, floating sub-heading word, keyword heading word, organism supplementary concept word, protocol supplementary concept word, rare disease supplementary concept word, unique identifier, synonyms] AND exp drug resistance, bacterial/or beta-lactam resistance/or cephalosporin resistance/or penicillin resistance/or ampicillin resistance/or methicillin resistance/or chloramphenicol resistance/or exp drug resistance, multiple, bacterial/or kanamycin resistance/or tetracycline resistance/or trimethoprim resistance/or vancomycin resistance/ OR (resistan* adj3 antibiotic*).mp. [mp = title, abstract, original title, name of substance word, subject heading word, floating sub-heading word, keyword heading word, organism supplementary concept word, protocol supplementary concept word, rare disease supplementary concept word, unique identifier, synonyms] OR ((Microbial* or anti-microbial or antibiotic* or beta-lactam or cephalosporin* or penicillin or tetracycline or trimethoprim or vancomycin or fluoroquinolone*or quinolone* or carbapenem* or teicoplanin* or aminoglycoside* or colistin*) adj3 resistan*).mp. [mp = title, abstract, original title, name of substance word, subject heading word, floating sub-heading word, keyword heading word, organism supplementary concept word, protocol supplementary concept word, rare disease supplementary concept word, unique identifier, synonyms].

**Fig. 1:**
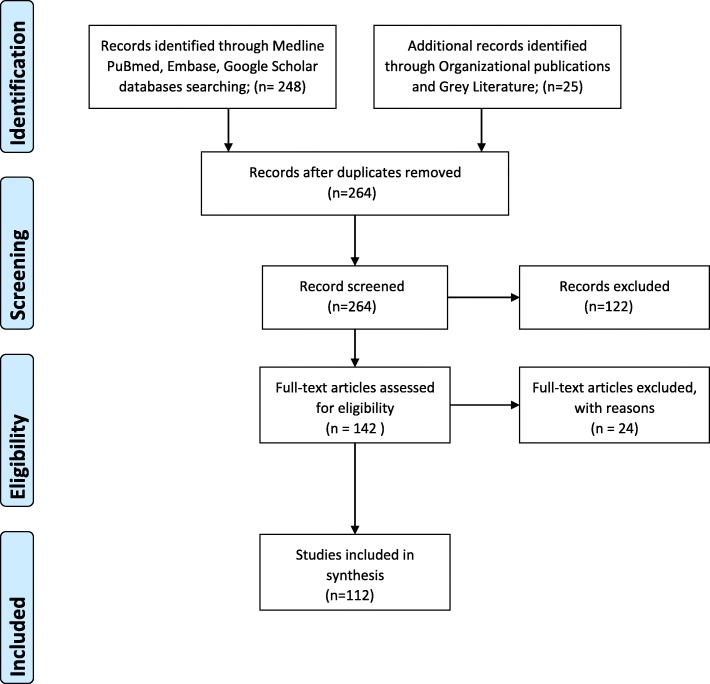
Search strategy and eligibility criteria

After finalizing Medline strategy, the search terms were appropriately adapted into the three other databases. The obtained results were screened and studies were excluded if their primary objective was not solely the evaluation of the burden of surgical site infection. Further reading and screening articles showed that it is not possible to quantify the burden of SSI if the accuracy and reliability of data is questionable due to gaps in surveillance and epidemiology methods and it was also not possible to discuss AMR prevention without further highlighting the gaps in current SSI infection and prevention practices. Further search included articles through gray literature and organizational publications (i.e. National Institute for Health and Care Excellence, Centers for Disease Control and Prevention, World Health Organization and European Center of Disease Prevention and Control). Further studies were identified by examining the reference lists of all included articles (Fig. 1).

## Results

### Economic burden of SSI

Surgical site infection is the leading cause of substantial burden worldwide [15–19]. It is the third most costly type of healthcare-acquired infection (HAI) with an estimated cost of US $20,785 per patient case [20]. The current annual costs to the health-care system amounted to billions of US$ has doubled since 2005 [20, 21]. The economic burden of SSIs is associated with direct medical costs related to prolonged hospitalization [22–25], intensive care units (ICU) stay [26], reoperation [27], surgical techniques [28], hospital readmission [29, 30], and consumption of medical resources [31]. These are attributed to investigation, diagnostic tests [13], medical staff namely skilled surgeon’s fees [28], operative costs [13], antibiotic prophylaxis [6], and treatment costs [11, 22–25, 32], in addition to the for-profit or not-profit nature of healthcare system services [28]. Indirect costs attributable to SSI are the increased risk of morbidity and mortality estimated two to eleven times greater in patients with SSI compared with non-infected patients [33–36], the decrease in the patient quality of life [37], absenteeism from work and loss of earnings during recovery [13]. Several studies consistently demonstrated the profound impact of SSI on the length of hospital stay [9, 11, 18, 25, 27, 29, 38, 39] with the number of hospitalization days varying per country, type of surgery [13], patient age and co-morbidities [40] whether associated with nosocomial infection [18] in addition to the presence of a prosthetic implant [41, 42]. The majority of the studies considered the increased costs of SSI relative to non-infected patients [23, 27, 30, 37, 43, 44] but very few [36] have evaluated the costs associated with infections due to resistant compared with susceptible bacteria. SSI due to AMR are difficult to treat and may represent a great challenge complicating further the clinical and economic outcomes of the disease [36, 45–52].

#### The concept of economic burden of SSI from different perspectives

In times where healthcare expenditure continues to climb and resources are limited, cost savings and shifting resources use from treating toward preventing infection, is an important goal. Current strategies, focusing only on the costs of SSI to improve the quality of care, are providing a myopic view of the real cost associated with infection in general and with SSI in particular. That is the wider cost of not having an effective antibiotic to prevent or treat the infection. Studies have demonstrated that the concept of cost savings varies according to the chosen perspective.

From the economists perspective, the costs associated with SSI treatment are viewed as an “opportunity cost” that deprive hospitals from using the allocated financial resources elsewhere [13] (i.e., investing in quality improvement plans). However, recent publications have challenged this paradigm [53, 54]. Rauh et al. claims that quality improvement may enhance hospitals profitability but will not drastically solve the fixed hospital costs dilemma [54]. It is the rigid cost structure that is relatively insensitive to changes in resources use, so clinical improvement will generate additional capacity to treat more patients but will not lead to bottom line savings [55]. So basically, understanding the cost layers in the healthcare system will provide management with a framework to target changes.

From the hospital perspective, taking preventive measures to avoid SSI and reduce readmission rate and hospital length of stay is thought to be a “top priority” [31], ultimately resulting in cost savings. Some studies show that accounting for these proxy to demonstrate cost savings is “illusory” [55, 56], may lead to bias and result in disputed outcomes [55, 57].

Furthermore, since the cost-effectiveness of some of these proposed measures is not yet demonstrated [58–60], then the hospitals may be allocating a higher budget and making less profit to potentially avoid these complications in the absence of incentives proving their clinical effectiveness. Therefore, cost savings through preventing SSI may be questionable unless the undertaken measures to fulfill this goal are evidence-based and part of the hospital strategic quality improvement plan.

From the payer perspective, there is a high financial benefit when SSI are avoided because they are linked to higher average payment to hospitals [31]. Variable strategies have been undertaken to give the hospitals the drive needed to reduce SSI. However, the current system of reimbursement may provide a financial disincentive to their reduction [61]. Certain strategies like financial penalties or excluding HAI in tariff have backfired through hospitals underreporting and reluctance in openly sharing SSI incidence results openly. Rather, it is proposed that it would be more productive to develop a system based on transparent reporting, financial reward, innovation, and inciting physician’s engagement [62]. Additional suggestions would be payers bundling the average costs of complications into the base diagnosis-related group (DRG) payment or limiting the hospital ability to recode retrospectively into higher paying DRG which may give the hospitals the incentive needed to avoid complications [61].

From a societal perspective, the magnitude of the economic burden of SSI might not be known if ascertainment is left solely to the index hospital’s information systems [13]. In monetary terms, cost savings from this perspective means avoiding indirect costs incurred by the patient through absenteeism from work and out -of- pocket payments to treat SSI infections. It also means the cost of avoiding pain and suffering and the negative effect on the quality -of -life but most importantly it means the cost needed to prevent antimicrobial resistance associated with SSI.

### Methods for evaluating the economic burden of SSI

The global variability of healthcare systems, financial structures, currencies, local epidemiologic data, and resistance patterns have limited the generalizability and the comparability of the economic evidence between countries [13]. This has highlighted the urgent need for high-quality studies using a standardized methodology for the evaluation of the economic burden of SSI [6, 63]. Literature review has shown that the major limitations in these studies are mainly related to (1) the uses of different definitions to classify SSI [7] and to the inability to follow-up with patients long enough post-surgical discharge [64]. Across the literature, the characteristics of population and subgroups analysis may differ. (2) Stratifying patients is crucial especially by age groups and underlying co-morbidities. Most of the studies rarely consider the surgical cases complications in pediatric population, known to be at higher risk of SSI and have different pathogens patterns [6]. Limitations in the methods of economic evaluation may also be related to (3) the location and settings were the study was conducted (i.e. Studies grouped into the same surgical specialty may not be comparable due to differences in operating theater conditions and surgical procedures [13, 63]). Some studies assigned the development of SSI to multiple or unspecified surgeries which can be a source of bias, limiting the comparability of data. In addition, it is highly recommended to account for differences in the effectiveness of antimicrobial stewardship programs, preoperative prophylactic strategies, treatment failure [63], infection control practices, and antimicrobial susceptibility testing across countries and settings. (4) Description of the study perspective and how it relates to the allocated costs is important. Literature review shows that studies were undertaken from different perspectives mainly the hospital and the payer perspective, accounting for the direct costs of treating SSI, and rarely considered the costs incurred from the patient perspective. Most importantly, some studies did not explicitly state the perspective and none have evaluated the wider impact on society and included the indirect costs, e.g. costs of pain, suffering, and loss of productivity [65]. (5) Comparators included across the literature considered patients with SSI versus uninfected patients [66]. It is argued that such comparison may lead to the overestimation of costs [67] mainly because the treatment of infection will increase the costs [57] especially if the causative agent is an AMR and patients may be at higher risks of additional co-morbidities leading logically to an extra incurred cost [67]. In order to minimize bias in quantifying the burden of SSI treatment, it is suggested to compare cases of SSI due to resistant- with cases due to susceptible -bacteria. (6) It is also noted that the time horizon is not considered consistently and may not capture all data. Since most cases of SSI occur post-discharge, some patients are not readmitted to the indicated hospital, or there may be difficulty following up the patient especially in LMICs [68]. (7) Discount rates if warranted and relevant cost components were either omitted or not clearly stated, including the incremental cost, discounting, and the results of sensitivity analysis [6, 63]. (8) Description of outcomes as the measure of benefit in the economic evaluation and their relevance to the type of analysis performed is highly recommended. The three most common economic evaluation tools are cost-effectiveness analysis, cost–utility analysis, and cost–benefit analysis; they differ in the nature of the measured consequences. Of note, the cost-of-illness analysis does not measure the outcomes but only the related costs of the disease. This type of study is considered a baseline to inform health-economic analysis. Literature review shows the uses of different study designs [53, 65, 69], and inappropriate allocated type of health-economic analysis. (9) Sources of data and methodology of data collection can be an important source of bias especially if not explicity described, if single-centered and collected retrospectively from hospital databases regardless if generated from high or LMICS. (10) SSI is a time-dependent exposure. However, time-dependent bias has been recognized as a problem in analyzing HAI infection data, and the appropriate type of analysis is subject of debate [70]. (11) A detailed description of the analytical method should be clearly stated including methodology of dealing with skewed, missing, or censored data, adjustments made, handling population heterogeneity and uncertainty, in addition to the assumptions and the model used if applicable [71].

### Reported gaps in SSI data gathering

#### Gaps in epidemiologic data

SSI is considered the second most common type of HAI in Europe and the USA. In low-to-middle-income countries (LMICs), data shows that one in ten people undergoing surgery acquire HAI [68, 72, 73]. It is estimated that SSI rates in developed countries vary between 1.2 and 5.2% while in developing countries, the pooled incidence is 11.8% per 100 surgical procedures [12]. Current figures may likely be underestimated because most data arise from hospital settings while around half of SSI cases become evident post-discharge [74]. In-hospital SSI estimates may not be reliable even in high-income countries since very few hospitals can afford to allocate time, budget, and human resources or because of the limited expertise in study design, data collection, or interpretation [9–11]. Other causes may be due to the fact that current surveillance reports may lack generalizability and comparability of data, they may be non-comprehensive to all types of surgeries, and not specific to the classification of infection (e.g., clean, contaminated, dirty). If SSI rates are to serve as a quality indicator and comparison benchmark for healthcare facilities, countries, and the public [5], there is an ongoing need for well-designed global surveillance system and high-quality studies that use a common approach to SSI definition, patient selection, determination of endpoints, and follow-up [13].

#### The need for standardized definitions of SSI

Standardizing the SSI definition is a challenge that requires a multidisciplinary expertise and allocation of time and resources. A systematic review by Bruce et al. identified 41 different definitions for SSI addressed in the literature among which very few were standardized and set by multidisciplinary groups [7, 75, 76]. SSI definitions are based on multiple factors such as site of infection and type of incision, presence of purulent discharge, clinical signs and symptoms, or physician diagnosis in a specific surveillance population, and laboratory results [16]. The Center of Disease Control and Prevention (CDC) [8, 77] refers SSI to “an infection that occurs after surgery in the part of the body where the surgery took place. Surgical site infections can sometimes be superficial infections involving the skin only. Other surgical site infections are more serious and can involve tissues under the skin, organs, or implanted material”; other definition by the ECDC [78] consider SSI as “an infection that occurs within 30 days after the operation and involves the skin and subcutaneous tissue of the incision (superficial incisional) and/or the deep soft tissue (for example, fascia, muscle) of the incision (deep incisional) and/or any part of the anatomy (for example, organs and spaces) other than the incision that was opened or manipulated during an operation (organ/space)”. In limited resources settings, the World Health Organization (WHO) [68] recommends to define SSI based on clinical signs and symptoms given the lack of quality microbiology laboratory support. The variability of SSI definitions and the methods used for the detection of infection should be accounted for when comparing evidence from different studies. Inconsistent application of definitions across all sites and time periods can generate poor data resulting from SSI surveillance [68, 79] which can potentially lead to underreporting of the disease, and invalid inter-country and inter-network infection rate comparisons and benchmarking [6, 79].

#### Gaps in SSI surveillance methodology

The need to develop a surveillance program for SSI is well recognized since the late 1960s . This proposition is credited to Dr. Cruse and his team who argued that retrospective data are not reliable, because hospital records are inaccurate for studies of SSI. They proposed a prospective wound surveillance [74, 80] currently considered the gold standard for an efficient surveillance strategy [81]. In developed countries, SSI surveillance is either mandatory or voluntary-based while in developing countries, data is scarce, primarily single-centered, hospital-based, especially in Asia, South America, and Africa [33]. Hospital-based surveillance is likely to underestimate the true rate of SSI, a problem that is exacerbated by the increasing trend toward shorter lengths of post-operative hospital stay and 1-day surgery [82]. Implementing a system that enables the identification of SSI cases post-discharge generates high-quality data; however, there are many challenges and practical difficulties in the community settings limiting the accurate and reliable identification of SSI cases and thus the generation of valid data [83]. On the other hand, a network-based surveillance may lead to various impact on SSI rates. Some studies report a positive outcome after participation in a network [82, 84, 85] while others report no changes [86]. It is argued that bias related to network-based surveillance methodologies can be avoided by adding hospitals to the network according to their year of participation [87] or stratifying SSI rates by surveillance time- to -operation in consecutive 1-year periods using the first year of surveillance as a reference [88]. However, till date, there is no gold standard method for post-discharge surveillance [89] nor an ideal method of surveillance design or implementation [90] nor a universally adopted cut-off length of surveillance. The CDC suggests a shortened period of 90 days post-discharge in order to avoid delayed feedback; however, this protocol is not always accounted for and depends on the type of surgical procedure being studied [91]. Choosing the outcome indicator is also subject to debate. Literature review shows that the most common outcome indicator is the cumulative SSI incidence also known as SSI rate. Some authors consider that reporting SSI using prevalence methods is considered less reliable and argue that the incidence density of in-hospital SSI is a more suitable choice by taking into account different lengths of hospital stay and different post-discharge surveillance methods. Accounting for the variations in case-mix and stratification of patient characteristics, choosing the appropriate risk-adjustment index is essential in order to improve the validity of comparisons [92, 93]. Reliable microbiology support is an essential component of SSI surveillance. However, clinical diagnosis of SSI can be made without microbiological confirmation, an approach that may be considered acceptable in countries with limited resources; it should be noted that this method can give an estimate of the overall rates of SSI in general but not the specific rates of bacterial resistance associated with SSI especially those occurring in LMICs, an area considered highly endemic [94].

#### SSI due to antimicrobial resistant pathogens

Resistance patterns of bacteria associated with SSI vary globally depending on the region, local epidemiology reports, and methodology of susceptibility testing. SSI treatment is becoming very complex and challenging [45, 46] due to bacterial resistance. The mainstay of adequate therapy is the early diagnosis of SSI and microbiological diagnostics [91]. Identification of the resistance patterns among SSI cases is crucial [95, 96] in order to avoid the misuse and abuse of antibiotics especially broad-spectrum drugs adding to the economic burden of the disease [56]. Studies have shown differences in the virulence of bacteria among outpatient compared with inpatient settings where inpatients population had a higher number of resistant organisms causing SSI [46, 97]. Most of the data comes from high-income countries where multidrug-resistant *Escherichia coli* and *Staphylococcus aureus* [46] are the most frequently reported isolates. Some studies report high incidence of gram-negative bacteria depending on the type of surgery being studied while other highlight the increased incidence of MRSA isolated from surgical sites [98]. However, despite scarce reports on the rates of resistant bacteria causing SSI especially from LMICs, studies evaluating the economic burden of SSI related to these pathogens are needed [6].

### Effectiveness of infection control and prevention strategies

The ultimate aim of preventing SSIs is to secure patient safety while decreasing the rate and burden of infection [99, 100] especially those due to AMR bacteria. Recently, the CDC [101], the WHO [12, 99], and the American College of Surgeons and Surgical Infection Society [102] published their guidelines for the prevention of SSI. These guidelines are intended to provide updated evidence-based recommendations from targeted systematic review [101] of the best evidence to prevent SSI. As a result, surgeons are given guidance about strong recommendations practices while they are left with no recommendations if the level of evidence is low to very low-quality with uncertain trade-offs between the benefits and harms [103]. These guidelines should be implemented as part of a comprehensive surgical quality improvement program using multimodal strategies [9, 64, 99, 100]. An unresolved issue/no recommendation level highlights the current gaps in research and the need for powered, well-designed randomized trials that addresses these issues especially in LMICs [64, 100, 101, 103]. This also means that some of the current practices considered an integrated part of the quality improvement plan may be consuming tremendous amount of time and resources potentially without evidence-based benefit adding to the burden of the SSI. Research gaps in the prevention of SSIs also extend beyond the current heterogeneous practices to a more crucial serious threat that is the prevention of SSI due to AMR bacteria [94].

## Discussion

Quantifying the economic burden of SSI is difficult and challenging in the absence of validated method to avoid bias and enhance generalizability of findings [104]. Literature search showed that most articles evaluating the costs of SSI considered the payer or hospital perspectives and compared SSI cases with no infection cases with very few exceptions considering SSI due to resistant bacteria [16, 36, 105, 106]. In an era where antibiotic resistance is affecting the world sustainable development [107], the optimal way to avoid bias in quantifying the burden of SSI is to consider the bigger impact of SSI due to resistant—compared with SSI due to susceptible—bacteria from the society perspective taking into account that infection is a time-dependent variable [69]. Estimating the burden of SSI is not only a budget issue or a public health issue, it is a global need to assess how health resources are spent, and to points out if expenditures are justified in terms of efficiency and effectiveness and most importantly how they are directly or indirectly affecting the world sustainable development. Literature search showed that we should start with continuous consistent global surveillance (Table 1), with a unified definition of SSI to allow comparability and extrapolation of findings. It may seem that this is the work of researchers and epidemiologists or may only be the government responsibility through health policies but in fact there are multiple other stakeholders including surgeons, other healthcare workers, the patient and family, and more broader, the society. It all starts in the operating room and depends on the type of surgery, the surgical procedure and on the effectiveness of practices to prevent SSI. It also extends to the applicability of infection control and prevention strategies during hospital stay and for a specific period after discharge, on the patient and family knowledge about the risks of SSI and related prevention strategies.

**Table 1:** Review of the suggested protocols for surgical site infection

Core value	Dedication, commitment, consistency, and leadership support
Fundamental	The same definition of SSI should be used across all sites and time periods
	In LMICs: definitions based on clinical signs and symptoms should be prioritized
Stakeholders	Government, society, patient, patient family, hospitals, and payers
Surveillance methods	Direct, prospective in-hospital and post hospital discharge
	In LMICs: possible mobile phone contact
Surveillance duration	Continuous surveillance of SSI rates per patient case and per surgical procedure
	In LMICs: At least 3 to 6 month
Patient follow –up	In-hospital
	30-days or up to 90 days post-discharge
	One year for surgical procedures that requires an implant
Surveillance team	Core team: surgical staff, theater staff and IPC staff
Surveillance team qualifications	Highly trained on surveillance method
	High level of competency for data management and analysis
	Basic background in epidemiology, microbiology, and communicable diseases
Surveillance protocol	Detailed written plan including elements of the surveillance process integrated into a comprehensive infection control risk assessment process
	Training materials and information sheets
	Detailed method of data validation
	Constant intensity of surveillance for an area of interest
Data	Detailed patient inclusion and exclusion criteria
	Stratifying by patient characteristics^a^
	Date of onset of infection, isolate results, antibiotic code, antimicrobial susceptibility testing results; microorganisms and antimicrobial resistance data
Data sources	Medical records and human resources records
	Financial services and Information services
	Ancillary service reports; admission diagnoses reports; administrative/management reports; public health reports; marketing reports
	Surgical database
	Other sources: quality/utilization management; risk management; community agencies; occupational/employee health; communication with caregivers
Data entry	Preferably electronic support previously tested for accuracy and reliability
Data collection tools	Hospital size, type, location, code, surveillance period start
	Post-discharge surveillance: READM; REPSURG; REPGP; REPPAT; ICSURG; ICGP; CPAT
Data analysis	Present risk-adjusted SSI incidence; crude estimates; NNIS risk index
Ethical issues	Patient, hospital, and unit confidentiality
	A pre-discharge patient education and engagement with a signed assent

READM = detection at readmission (= passive post-discharge surveillance): patient is readmitted with SSI, often because of the SSI; REPSURG = reporting on surgeon’s initiative: surgeon actively reports post-discharge infections detected at outpatient clinic or private clinic follow-up to the hospital surveillance staff, e.g., using standardized forms, web-based system, e-mail, or telephone; REPGP = reporting on GP’s initiative: general practitioner (GP) reports post-discharge infections detected at follow-up consultation to the hospital surveillance staff, e.g., using standardized forms, web-based system, e-mail or telephone; REPPAT = reporting on patient’s initiative: e.g., form send to hospital surveillance staff; ICSURG = obtained by IC staff from surgeon: the hospital surveillance staff—usually infection control (IC) staff—obtains information from surgeon using telephone, additional questionnaire, visit to surgeon or patient chart review; ICGP = obtained by IC staff from GP: hospital surveillance staff obtains information from general practitioner using telephone, additional questionnaire or visit; CPAT = obtained by IC staff from patient: hospital surveillance staff obtains information from patient using telephone or additional questionnaire References: [5, 68, 78, 90]

^a^Age; sex; type of surgical procedure; whether elective or emergency surgery; the American Society of Anesthesiologists (ASA) score; timing and choice of antimicrobial prophylaxis; preoperative skin preparation; other indicators, e.g., protocol for intensive perioperative blood glucose control used and blood glucose levels monitored; implant in place; multiple operations during the same session or not; endoscopic procedure or not; duration of the operation; and wound contamination class; site of infection and type of SSI (superficial, deep, organ/space); number of OR openings; microbiology confirmation; outcome from hospital; patient discharge date; readmission date

Based on this review and the results of included studies, the following actions are recommended to tackle the reported gaps in:

1. The methodology of SSI surveillance (Table 1) [5, 68, 78, 90]
  - Set a unified comprehensive definition of SSI
  - Design a standardized SSI surveillance system that allows global, regional, and national benchmark and comparability of data
  - Determine the incentives and support needed for a valid data gathering
  - Set a focused priority list of resistant pathogens causing SSI as guidance for research studies
  - Assess and address the challenges of appropriate and reliable data gathering methodology in developed as well as in developing countries and evaluate the barriers and limitations in resources and expertise
  - Report consistently the surveillance data gathered in-hospitals and post-discharge
  - Suggest and validate open access training materials for accurate data gathering, data entry and analysis
2. The methodology of quantifying the burden of SSI (Table 2) [15, 68]
  - Design high-quality prospective studies to quantify the burden of SSI and consider infections due to resistant -compared with susceptible- bacteria pathogens.
  - Consider matched cohorts and take into account the site and type and modality of surgical intervention, the classification of surgery, patient factors (i.e., age, underlying co-morbidities), surgical theater factors and IPS, physician factors, and follow-up period.
  - Choose an appropriate methodology to evaluate the economic burden of SSI and take into account confounding factors and biases especially time dependence bias [69]
  - Address the wider impact and consider the perspective of society
3. The research studies of SSI (Table 2) [15]
  - Tackle the economic and clinical impact of SSI and SSI prevention strategies with a special focus on pediatric and geriatric population
  - Fill the research gaps in LMICs taking into consideration the resources limitation and explore the gaps and the barriers in data extrapolation and comparability in high income countries
  - Consider evaluating the cost-effectiveness and the cost-utility of SSI prevention strategies

**Table 2:** Gaps in research for the prevention of SSI

Main topic	Recommended
Parenteral antimicrobial prophylaxis	Selection of the most appropriate antibiotic according specific to different surgical procedure especially cardiac and vascular surgeries
	The optimal timing of preoperative SAP according specific to different surgical procedure [15]
	The optimal doses, intra-operative dose adjustments and re-dosing protocols of antibiotics [15]
	The effect of weight-adjusted parenteral antimicrobial prophylaxis dosing on the risk of SSI [15]
	The effect of prolonged antibiotic prophylaxis on the microbiome
Nonparenteral antimicrobial prophylaxis	The effect of intra-operative antimicrobial irrigation
	Comparisons between the most commonly-used irrigation practices
	Evaluation of the practices of soaking prosthetic devices in antimicrobial solutions before implantation for the prevention of SSI
	Assessment of the need for applying an autologous platelet-rich plasma for the prevention of SSI [15]
	Evaluation of the use of Antimicrobial-coated sutures for the prevention of SSI [15]
	Comparison between the antimicrobial coated and non-coated sutures using the same type of suture material, including non-absorbable sutures
	Evaluation of the use of antimicrobial dressings applied to surgical incisions after primary closure in the operating room for the prevention of SSI [15]
	Investigation of potential effects and adverse effects related to the use silver-containing dressings especially in orthopedic and cardiac surgery
	Comparison between the uses of opaque dressings and transparent ones in terms of postoperative visual examination and the duration of keeping the primary dressing in place
Glycemic control	The optimal hemoglobin A1C target levels for the prevention of SSI in patients with and without diabetes [15]
	The optimal route of insulin administration and the optimal timing and duration of perioperative glycemic control [15]
	The optimal duration of continued postoperative glucose control
	Comparison of different blood glucose target levels to define the optimal level with minimum risk of hypoglycemia
Normothermia	Comparison and selection of the optimal warming device and the proper timing and duration of warming practices
	The optimal timing, duration and limit of normothermia [15] and determine the target temperature
Perioperative oxygenation	The administration of increased FIO2 via endotracheal intubation during only the intra-operative period in patients with normal pulmonary function undergoing general anesthesia [15]
	The optimal target level, duration, and delivery method of FIO2 for the prevention of SSI [15]
	The administration of increased FIO2 via face mask during the perioperative period in patients with normal pulmonary function undergoing general anesthesia without endotracheal intubation or neuraxial anesthesia [15]
	The administration of increased FIO2 via face mask or nasal cannula during only the postoperative period in patients with normal pulmonary function [15]
	The optimal target level, duration, and delivery method of Fio_2_ [15]
	Investigations of the benefit of post-extubation hyperoxemia, including different durations, concentrations and oxygen administration routes
	The effect of hyperoxygenation on the incidence of SSI
	The consequences of the use of a higher concentration of narcotics, hypnotics or inhalational agents or muscle relaxants
Antiseptic prophylaxis	The optimal timing of the preoperative shower or bath, the total number of soap or antiseptic agent applications, or the use of chlorhexidine gluconate washcloths [15]
	Cost-effectiveness analyses to examine timing and duration of bathing in different types of surgery and wound classes, especially in LMICs
	Comparison of different antiseptic agents to each other and to plain soap for preoperative bathing
	Assessment of the effect of soap or antiseptics on the skin microbiome
	Evaluation of the effect of chlorhexidine gluconate (CHG) in reducing SSI and their cost implications
	The need for a antimicrobial sealant immediately after intraoperative skin preparation [15]
	The need of plastic adhesive drapes with or without antimicrobial properties [15]
	The practice intraoperative irrigation of deep or subcutaneous tissues with aqueous iodophor solution [15]
	The practice of intraperitoneal lavage with aqueous iodophor solution in contaminated or dirty abdominal procedures [15]
	The repeat application of antiseptic agents to the patient’s skin immediately before closing the surgical incision [15]
	Comparison of specific preparations containing CHG, PVP-I and other antiseptics in alcohol-based and other solutions
Blood transfusion	The effect of blood transfusions on the risk of SSI in prosthetic joint arthroplasty
Perioperative discontinuation of Immunosuppressive agents	The effect of systemic corticosteroid or other immunosuppressive therapies on the risk of SSI in prosthetic joint arthroplasty [15]
	The optimal time between discontinuation of immunosuppressive
	The optimal dose of the various immunosuppressive therapy agents including new ones with regards to the SSI rate
	The use and timing of preoperative intra-articular corticosteroid injection on the incidence of SSI in prosthetic joint arthroplasty [15]
Anticoagulation	The use of venous thromboembolism prophylaxis on the incidence of SSI in prosthetic joint arthroplasty [15]
Orthopedic surgical space suit	The use of orthopedic space suits or the health care personnel who should wear them for the prevention of SSI in prosthetic joint arthroplasty [15]
Biofilm	The cement modifications and the prevention of biofilm formation or SSI in prosthetic joint arthroplasty [15]
	Prosthesis modifications for the prevention of biofilm formation or SSI in prosthetic joint arthroplasty [15]
	The uses of vaccines for the prevention of biofilm formation or SSI in prosthetic joint arthroplasty [15]
Decolonization with mupirocin for the prevention of *Staphylococcus aureus* infection in nasal carriers	Determination of the surgical patient population that should undergo screening for *S*. *aureus* carriage
	Determination of the timing and duration of mupirocin administration and bathing in surgical patients
	Investigating other agents for the decolonization of nasal S. aureus carriers scheduled for surgery
Screening for extended-spectrum beta-lactamase colonization and the impact on surgical antibiotic prophylaxis	Investigations of the tailored modification of SAP in areas with a high prevalence of ESBL-producing Enterobacteriacae, including patients known to be colonized with ESBL, is more effective in reducing the risk of SSI than no modification of the standard prophylaxis
	The effect of a routine screening for ESBL prior to surgery on the widespread use of broad-spectrum antibiotics pre-surgery in ESBL-colonized patients and the emergence of resistance in gram negative bacteria, especially carbapenem-resistant Enterobacteriacae
Mechanical bowel preparation and the use of oral antibiotics	Comparison of oral antibiotics and adequate intravenous prophylactic antibiotics vs. adequate intravenous prophylactic antibiotics only RCT focusing on laparoscopic procedures
Hair removal	Evaluation of the optimal timing and the most appropriate setting (ward vs. home) for the hair removal procedure when it is considered necessary by the surgeon
	The best and most acceptable methods of hair removal in settings with limited resources need to be investigated, including low-cost solutions
	Test evidence-based procedures on how to decontaminate clippers
	Studies with a focus on the use of clippers in LMICs
Surgical hand preparation	Comparison of the effectiveness of various antiseptic products with sustained activity to reduce SSI vs. ABHR or antimicrobial soap with no sustained effect
	Assessment of the interaction between products used for surgical hand preparation and the different types of surgical gloves, in relation to SSI outcome
Nutritional support	The impact of nutritional support in LMICs
	Investigating the benefit of other nutritional elements (for example, iron, zinc) and vitamins
	The optimal timing and duration of the administration of nutritional support in relation to the time of surgery
Maintenance of adequate circulating volume control/normovolemia	Identification of the most accurate and least invasive method of measuring normovolemia and assess its influence with regard to tissue oxygenation and normothermia
Drapes and gowns	Investigating the use of sterile disposable compared to sterile reusable drapes and surgical gowns in terms of SSI prevention
	Types of materials (including permeable and impermeable materials) and address environmental concerns (water, energy, laundry, waste, etc.)
	Investigating whether drapes should be changed during the operation and if this measure has an effect on SSI rates
	Investigating the potential benefits of these products
Wound protector devices	Comparison of single with double-ring WP devices and reporting adverse events related to the intervention
Prophylactic negative pressure wound therapy	Investigating the use of pNPWT for SSI prevention
	The identification the cost effectiveness of pNPWT in different groups of patients including those undergoing contaminated and dirty procedures
	The identification the optimal level of negative pressure and duration of application
Use of surgical gloves	Investigating the effectiveness of double-gloving compared to the use of a single pair of gloves would be welcome on SSI
Comparing different types of gloving to address the question of the optimal type of gloves to be used	Valuation whether a change of gloves during the operation is more effective in reducing the risk of SSI than no change of gloves are needed, including an assessment of the criteria for changing gloves during the surgical procedure
Changing of surgical instruments	Investigating the change of instruments prior to wound closure
Laminar airflow ventilation systems in the context of operating room ventilation	The effects of laminar flow in reducing the SSI rate, require a massive investment with a high sample size to have enough power to see a difference
	The impact of fans/cooling devices and natural ventilation on the SSI rate compared to conventional ventilation in order to evaluate whether these systems might be an alternative in resource-limited countries
Optimal timing for wound drain removal	The optimal timing for drain removal especially in orthopedic joint replacement and cardiac surgery and the effect on SSI
	Investigating the benefit of early drain removal in pediatric populations and among neonates

*LMICs* low–middle-income countries, *SAP* surgical antibiotic prophylaxis, *PK* pharmacokinetic, *PD* pharmacodynamics, *FIO*_2_ fraction of inspired oxygen, *CHG* chlorhexidine gluconate (CHG), *ESBL* extended spectrum beta-lactamase, *MBP* mechanical bowel production, *ABHR* alcohol-based hand rub, *pNPWT* prophylactic negative pressure wound therapy, *OR* operating room

## Conclusions

In an era of increased pressure for cost containment and alarming reports of the projected impact of AMR, quantifying the burden of SSI due to resistant bacteria can inform the governments and decision makers about the magnitude of the disease and provide incentives to invest in preventive strategies that tackles both the inpatient and outpatient settings. However, if efforts to reduce SSI are taken in isolation without a global alliance and data is still lacking generalizability and comparability, we may see the future as a race between the global research efforts for the advancement in surgery and the global alarming reports of the increased incidence of antimicrobial resistant pathogens threatening to undermine any achievement.

## Data Availability

Not applicable.

## Abbreviations

AMR: Antimicrobial resistance
CDC: Center of Disease Control and Prevention
DRG: Diagnosis-related groups
ICU: Intensive care unit
LMICs: Low-to-middle-income countries
MeSH: Medical subject headings
SSI: Surgical site infection
WHO: World Health Organization
